# Acute Effects of Inorganic Nitrate Intake on Brachial and Femoral Flow-Mediated Vasodilation, and on Carotid Artery Reactivity Responses: Results of a Randomized, Double-Blinded, Placebo-Controlled Cross-Over Study in Abdominally Obese Men

**DOI:** 10.3390/nu14173560

**Published:** 2022-08-29

**Authors:** Ellen T. H. C. Smeets, Ronald P. Mensink, Jordi P. D. Kleinloog, Peter J. Joris

**Affiliations:** Department of Nutrition and Movement Sciences, NUTRIM School of Nutrition and Translational Research in Metabolism, Maastricht University Medical Center, P.O. Box 616, 6200 MD Maastricht, The Netherlands

**Keywords:** inorganic nitrate, flow-mediated vasodilation, carotid artery reactivity, vascular endothelial function

## Abstract

Most trials on the effects of inorganic nitrate intake have focused on only one specific aspect of the endothelial cell response to a stimulus, thereby possibly missing other important effects. The aim of the present randomized, double-blinded, placebo-controlled cross-over study was therefore to investigate in eighteen healthy abdominally obese men (18–60 years, waist circumference ≥ 102 cm) acute effects of potassium nitrate on brachial and femoral flow-mediated vasodilation (FMD), and on carotid artery reactivity (CAR) to a cold pressure test. Participants received in random order a drink providing 10 mmol potassium nitrate (i.e., 625 mg of nitrate) or an iso-molar placebo drink with potassium chloride. Fasted and 4 h post-drink FMD and blood pressure measurements were performed. CAR responses were assessed at 4 h. Circulating nitrate plus nitrite concentration increased following nitrate intake (*p* = 0.003). Compared with placebo, potassium nitrate did not affect brachial (mean [95% confidence interval]: −0.2% [−2.5, 2.1], *p* = 0.86) and femoral FMD responses (−0.6% [−3.0; 1.7], *p* = 0.54). CAR responses were also not different (−0.8% [−2.5, 0.9], *p* = 0.32). Finally, changes in blood pressure and heart rate did not differ. No adverse events were observed. In conclusion, this trial did not provide evidence for effects of a single dose of inorganic nitrate on 4 h vascular endothelial function in abdominally obese men.

## 1. Introduction

A reduced nitric oxide (NO) bioavailability contributes to an impaired function of the vascular endothelial cell layer, which may eventually lead to the development of cardiovascular disease (CVD) [1,2]. A possible strategy to improve vascular endothelial function is by increasing the intake of inorganic nitrate, which increases NO bioavailability via the nitrate–nitrite–NO pathway [3,4]. Inorganic nitrate can be provided by nitrate-rich food products such as green leafy vegetables and beetroot [5,6] or by nitrate salts. Most well-controlled intervention studies investigating the effects of inorganic nitrate have however focused on only one specific aspect of the endothelial cell response to a stimulus. Thus, Jackson et al. [7] concluded from their meta-analysis that a single dose of inorganic nitrate improved the endothelial function of the brachial artery, as measured by endothelium-dependent flow-mediated vasodilation (FMD). However, the endothelial function can be assessed in other peripheral muscular arteries as well, such as the superficial femoral artery. Although FMD responses of the brachial and femoral arteries are both NO-mediated, responses to interventions of these two arteries may be different [8]. Recently, however, Walker and colleagues [9] reported that beetroot juice consumption also improved 2 h femoral artery FMD. As beetroot juice contains several other potentially beneficial bioactive components besides dietary nitrates, such as vitamins, phenolics, plant proteins, and carotenoids [10], it remains unclear if these effects are caused by the nitrate or by one of the other bioactive components. Finally, the endothelial function can also be examined in a central elastic artery by assessing carotid artery reactivity (CAR) to a cold pressure test [11]. Although the exact underlying mechanisms of the CAR to a cold pressure test are still unknown, CAR responses also provide useful information about future CVD events [12]. However, no trials have investigated the effects of inorganic nitrate on these responses. In addition, the use of different non-invasive vascular function measurements provides a more complete picture of the impact on endothelial function throughout the arterial tree. This randomized, double-blinded, placebo-controlled cross-over study in eighteen abdominally obese men, therefore, investigated the effects of a single dose of inorganic nitrate on brachial and femoral FMD, and on CAR to a cold pressure test.

## 2. Materials and Methods

### 2.1. Study Population

Twenty-three apparently healthy abdominally obese male volunteers were recruited through advertisements in local newspapers, flyers in universities, or among participants who had participated in earlier studies. Important inclusion criteria were: aged between 18 and 60 years, waist circumference ≥ 102 cm, stable body weight (weight gain or loss < 3 kg within the previous 3 months), no use of anti-hypertensive medication or drugs known to affect lipid or glucose metabolism, no diabetes or active CVD, and no participation in another trial during the past 30 days. All participants gave written informed consent before entering the study. The study was approved by the Medical Ethical Committee of the University Hospital Maastricht/Maastricht University (METC azM/UM) and executed between January and May 2021. The trial was registered online at ClinicalTrials.gov as NCT04700241.

### 2.2. Study Design

A randomized, double-blinded, placebo-controlled cross-over study was performed consisting of two test days, separated by a wash-out period of at least one week (see Supplementary Figure S1). On the week preceding each test day, participants were asked not to use antibacterial mouthwash or toothpaste, chewing gum, or a tongue scraper. In addition, study participants were asked not to consume nitrate-rich food products a week prior to the test days. Moreover, participants were asked not to perform any strenuous physical activity on the two days preceding each test day and not to consume any alcohol on the day prior to each test day. Throughout the whole study period, participants were also kindly requested not to change their habitual food intake or physical activity levels.

Participants came to the Metabolic Research Unit Maastricht (MRUM) by car or by public transport after an overnight fast (from 20.00 h) to standardize measurements. After insertion of an intravenous cannula, the participant had to rest for 15 min in supine position after which fasting vascular measurements were performed. After completion, a fasting venous blood sample (T0) was collected. Thereafter, the participants had to consume either a nitrate-rich or placebo drink. Subsequent blood samples were collected 60 min (T60), 120 min (T120), 180 min (T180), 210 min (T210), 240 min (T240), and 330 min (T330) after drink consumption. Moreover, exogenous insulin (160 IU, Novorapid, Novo Nordisk, Mainz, Germany) was administered as nasal spray to assess insulin action on the brain 150 or 180 min after consumption of the drinks as described elsewhere [13].

### 2.3. Investigational Drinks

Investigational drinks were prepared by an independent person on the morning of a test day. The nitrate-rich drink consisted of 10 mmol (i.e., 625 mg nitrate) potassium nitrate (Merck KGaA, Darmstadt, Germany) solved in 30 mL of water. The placebo drink was an iso-molar potassium chloride, also solved in 30 mL of water (Merck KGaA, Darmstadt, Germany). Except for the independent person, participants and study personnel were blinded to the treatments as drinks were similar in both appearance and volume.

### 2.4. Anthropometric and Vascular Measurements

Body height was measured during the screening visit using a wall-mounted stadiometer. Before the start of each test day, body weight was measured without shoes and heavy clothing. Furthermore, waist and hip circumferences were measured.

Office brachial systolic blood pressure (SBP), diastolic blood pressure (DBP), and heart rate (HR) were measured in supine position before the start of the vascular endothelial function measurements using a semi-continuous blood pressure monitoring device (Omron M7 IntelliSense^TM^, Omron, Hoofddorp, The Netherlands). The first measurement was discarded and the average of the last three measurements was reported. Vascular function measurements were performed in a quiet and darkened room with a stable room temperature of 22 °C. All vascular measurements were performed by the same sonographer (E.S.).

Fasting and 4 h post-drink brachial and superficial femoral FMD measurements were performed using a MyLab^TM^ ultrasound machine with a 13-MHz linear transducer (MyLabTM-Gamma, Esaote, Maastricht, The Netherlands). The brachial artery was scanned above the elbow, whereas the femoral artery was scanned above the knee in longitudinal direction to ensure proper visualization of the artery before the start and during the measurement. Measurements were performed in B-mode with Doppler to assess continuous artery diameter and velocity profiles as recommended [14]. After a 3 min reference period, the pneumatic cuff placed distal (i.e., below the elbow or knee) to the ultrasound probe was inflated to 200 mmHg. After 5 min, the cuff was deflated to induce reactive hyperemia. The echo images were analyzed offline to determine diameter and velocity profiles over the entire measurement period with a custom-written Matlab program using automated edge-detection and wall tracking (MyFMD V15.06, Prof. A.P.G. Hoeks, Department of Biomedical Engineering, Maastricht University, Maastricht, The Netherlands). The FMD was calculated as the percentage change in post-occlusion peak brachial or femoral artery diameter relative to the baseline diameter. In addition, to correct for potential changes in velocity stimulus, the FMD corrected for peak velocity (pFMDv) was calculated by dividing the FMD by the percentage change in post-occlusion peak velocity flow [15]. Finally, the time needed to reach its peak diameter after cuff release (i.e., time to peak dilation) was calculated.

Carotid artery reactivity (CAR) to a cold pressure test was only assessed 4 h post-drink using the same echo-Doppler device. The left common carotid artery was scanned in the longitudinal direction to ensure proper visualization of the artery before the start and during the measurement. Similar to the FMD, measurements were performed in B-mode to assess artery diameter profiles. The cold pressor test consisted of a one-minute baseline period and three-minute immersion of the hand in a bucket of cold water (4.0 °C) with ice slush. The echo images of the entire 4 min period were also analyzed offline with the same software as for the FMD. The CAR response was quantified as the maximal percentage change in the cold-induced reaction in arterial diameter relative to the baseline diameter.

### 2.5. Biochemical Analyses

NaF plus Na_2_EDTA-containing 4 mL tubes (Becton Dickinson, Erembodegem, Belgium) were collected and centrifuged at 1300× *g* for 10 min at 4 °C immediately after sampling to obtain plasma. Furthermore, blood was collected in 8.5-mL serum STT-II advance tubes (Becton Dickinson) and placed for at least 30 min at 21 °C allowing the blood to clot. After clotting, the tubes were centrifuged at 1300× *g* for 10 min at 21 °C to obtain serum. Following centrifugation, plasma and serum were immediately portioned into aliquots, frozen into liquid nitrogen, and stored at −80 °C until analysis at the end of the study.

Fasting (T0) plasma glucose (Glucose HK CP, Horiba ABX, Montpellier, France) concentrations were measured in NaF plasma. Serum insulin (human-specific ELISA, Crystal Chem, Zaandam, The Netherlands), total cholesterol (CHOD-PAP method; Roche Diagnostic Systems), high-density lipoprotein cholesterol (HDL-cholesterol; precipitation method; Roche Diagnostics, Mannheim, Germany), triacylglycerol (TAG; GPO Trinder, Sigma-Aldrich Corp., St. Louis, MO, USA) with correction for free glycerol and plasma high-sensitivity C-reactive protein concentrations (hsCRP; CRP CP, Horiba ABX, Montpellier, France) were only measured at T0. In addition, serum nitrate plus nitrite concentrations (colorimetric nitrite/nitrate Griess assay kit, Sigma-Aldrich Corp., St. Louis, MO, USA) were assessed at T0, T60, T120, and T240. This assay determined total NO metabolite concentrations using the Griess assay [16]. Fasting LDL-cholesterol concentrations were calculated using the Friedewald formula.

### 2.6. Statistical Analyses

All data are presented as means and standard deviations (SD) unless stated otherwise. A total of eighteen volunteers was needed to detect a treatment difference in brachial artery FMD of at least 1.75%-point with an intra-subject variability of 2.30% [17], when an alpha of 0.05, a power of 80%, and a two-sided test were used.

Repeated measures ANOVA was performed to test for differences in fasting (T0) values. A linear mixed model with treatment and period as fixed factors was performed to assess differences in changes between drinks in FMD and blood pressure parameters. The participant was included as random factor and a random intercept was used. As changes were used as dependent variable, the intercept of the model represented changes over time. Differences in post-drink CAR responses were analyzed by linear mixed models with period as fixed factor and participant as random factor with random intercept. Changes in nitrate plus nitrite concentrations were analyzed by linear mixed models with random intercept. Period, treatment, time, and time × treatment were added to this model as fixed factors and participants as random factors. Post hoc tests with Bonferroni correction were performed when the interaction was significant. The level of significance was set at *p* < 0.05. Statistical analyses were performed using SPSS 23.0 software for Mac (SPSS Inc. Chicago, IL, USA).

## 3. Results

### 3.1. Study Participants

A total of 23 volunteers were screened and two men were excluded due to fasting serum total cholesterol concentrations above 8.0 mmol/L (*n* = 1) or contra-indications for one of the study outcomes (*n* = 1). The remaining 21 participants were randomized, but two participants dropped out before the start of the study and one after the first test day due to personal reasons. The CONSORT flow diagram of the volunteers screened, included, and analyzed in this randomized trial is shown in Supplementary Figure S2.

The median age of the eighteen volunteers who completed the whole study was 50 (interquartile range (IQR): 45–57) years. The participants had an average BMI of 33.5 ± 5.0 kg/m^2^ and their waist circumference was 117.3 ± 10.3 cm. No study-related (serious) adverse events were reported. Baseline characteristics, assessed during the screening visit are shown in Table 1.

### 3.2. Brachial and Femoral Flow-Mediated Vasodilation

**Figure 1:**
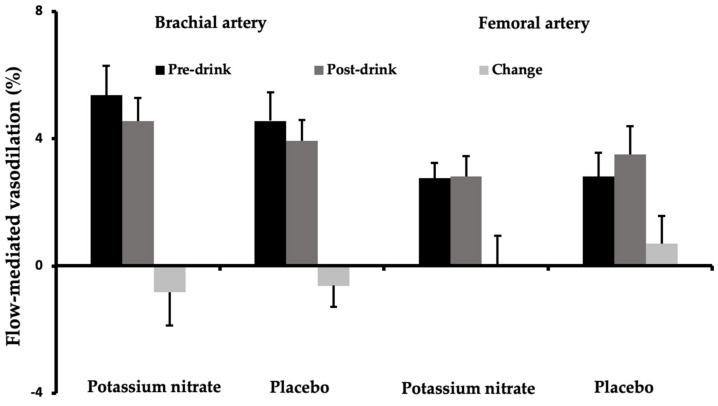
Mean brachial and femoral artery flow-mediated vasodilation (± SEM) responses before (pre-drink) and after (post-drink) the potassium nitrate and placebo drink (*n* = 18).

As shown in Figure 1, fasting brachial and femoral FMD responses did not differ between test days (*p* = 0.10 and *p* = 0.96, respectively). Moreover, changes in brachial (−0.8% vs. −0.6%, *p* = 0.86) and femoral (0.0% vs. 0.7%, *p* = 0.54) artery FMD responses were not significantly different between the nitrate salt and the placebo drink.

Fasting brachial and femoral artery diameters were also comparable between test days (*p* = 0.64 and *p* = 0.72, respectively, Table 2). A significant decrease in post-drink brachial artery diameters was found as compared with fasting values (*p* = 0.001, Table 2). However, changes in baseline diameters of the brachial (*p* = 0.34) and femoral artery (*p* = 0.73) did not differ between the nitrate-rich and placebo drink.

In line with FMD responses, fasting brachial and femoral pFMDv responses were not different between test days (*p* = 0.39 and *p* = 0.67, respectively). A significant decrease in post-drink brachial pFMDv values was found as compared with fasting values (*p* = 0.032). However, no significant differences in brachial and femoral pFMDv responses were found between drinks (*p* = 0.54, and *p* = 0.65, respectively). In addition, fasting flow responses were not different (*p* = 0.76 and *p* = 0.25, respectively). A significant decrease in post-drink brachial flow responses was found as compared with fasting values (*p* = 0.001). However, changes in baseline brachial (*p* = 0.45) and femoral flow responses (*p* = 0.78) did not differ between the nitrate-rich and placebo drink. Moreover, no differences in fasting brachial and femoral time to peak dilation were found (*p* = 0.28 and *p* = 0.37, respectively). Furthermore, changes were comparable between the potassium nitrate and placebo drinks for both the brachial (*p* = 0.14) and femoral artery (*p* = 0.74).

### 3.3. Carotid Artery Reactivity

**Figure 2:**
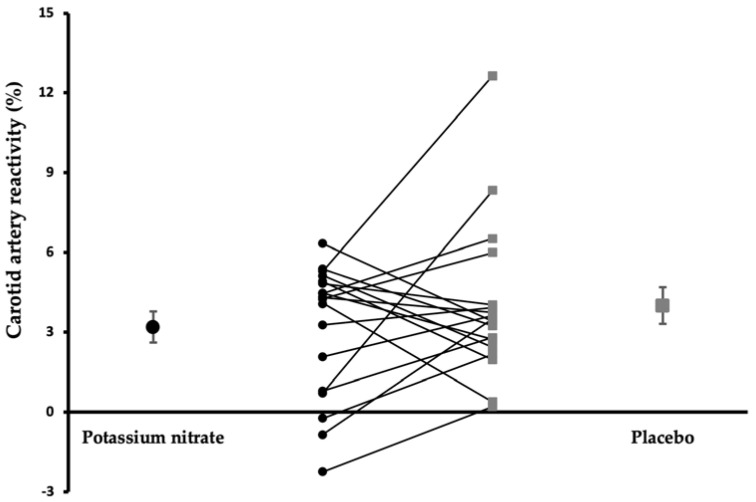
Mean (± SEM) and individual carotid artery responses to a cold pressor test after the potassium nitrate and placebo drink (*n* = 18).

CAR responses to a cold pressure test were assessed 4 h after consumption of the drinks. No differences in CAR responses between drinks were observed (3.2% vs. 4.0%, *p* = 0.32, Figure 2). However, baseline carotid artery diameters were significantly higher after the drink providing potassium nitrate as compared with placebo (*p* = 0.020, Table 2).

### 3.4. Blood Pressure

Fasting SBP, DBP, and HR values did not differ between test days (*p* = 0.93, *p* = 0.88 and *p* = 0.68, respectively). SBP (*p* = 0.009) and DBP (*p* = 0.001) increased over time, while HR (*p* < 0.001) decreased. However, changes in SBP (*p* = 0.76), DBP (*p* = 0.54) and HR (*p* = 0.35) did not significantly differ between drinks.

### 3.5. Post-Drink Glucose, Insulin, and Nitrate plus Nitrite Concentrations

Fasting serum total cholesterol, HDL- and LDL-cholesterol, TAG, hsCRP, and nitrate plus nitrite concentrations were comparable between test days (*p* > 0.05 for all values, Supplementary Table S1). Further, fasting plasma glucose and serum insulin concentrations did not differ (*p* = 1.00 and *p* = 0.21, respectively). Post-drink changes in glucose and insulin concentrations have been described elsewhere [13]. In brief, post-drink plasma glucose concentrations were significantly decreased from fasting concentrations, but no differences between drinks were found. Serum insulin concentrations did not change over time, while no differences were observed between drinks. Finally, serum nitrate plus nitrite concentrations significantly increased following the intake of potassium nitrate as compared with placebo (time × treatment, *p* = 0.003, Supplementary Figure S3). These concentrations were higher at T60, T120, and T240 after consumption of the inorganic nitrate drink (*p* < 0.001, for all time points).

## 4. Discussion

In this randomized, double-blinded, placebo-controlled cross-over study with abdominally obese men, the acute intake of potassium nitrate did not affect endothelial function, as assessed by brachial and femoral FMD, and by CAR to a cold pressure test.

Previously, beneficial effects have been reported on 1.5 and 2 h brachial FMD in healthy individuals following the intake of 8 mmol sodium nitrate (i.e., 500 mg of nitrate) [18] or 24 mmol potassium nitrate (i.e., 1488 mg of nitrate) [19]. Comparable effects were found after the acute consumption of beetroot juice providing about 500 mg of dietary nitrate [4]. Furthermore, Walker and colleagues [9] have found that beetroot juice with an average listed content of dietary nitrate of approximately 800 mg beneficially affected 2 h femoral FMD in healthy older adults. In the present study, a single dose of 10 mmol (i.e., 625 mg nitrate) of inorganic potassium nitrate was used, which is comparable to the amount of inorganic nitrate used in previous studies. In addition, Jonvik et al. [20] have shown that increases in both plasma nitrate and nitrite concentrations were comparable after the intake of a sodium nitrate drink and beetroot juice in healthy adults. Therefore, the use of nitrate salt is unlikely to explain the absence of an effect on endothelial function in the present study. However, a possible explanation for our findings is that in the present study abdominally obese men were included, whereas, in most previous studies, healthy adults with a normal body weight participated. Obesity may interfere with the enterosalivary nitrate–nitrite–NO pathway [21]. Obese individuals, who also have reduced FMD values at baseline [22], may thus be less responsive to the effects of inorganic nitrate. In fact, treatment effects were also not observed on 2 h brachial FMD responses following beetroot consumption (500 mg of nitrate) in CVD patients who also have impaired endothelial function [23]. Furthermore, previous trials [4,9,18,19] assessed 1.5 and 2 h FMD responses, while in the present study effects were measured 4 h after consumption of the drinks. Rodriguez-Mateos et al. [24] assessed hourly brachial artery FMD responses in healthy adults up to 4 h after a single dose of 8.0 mg/kg body weight of inorganic nitrate, which equals the amount provided in this study. Interestingly, the authors observed that 1 h brachial artery FMD was significantly improved, while effects on FMD responses gradually decreased after one hour and returned back to normal 4 h after nitrate intake. In agreement, Bahra and colleagues [25] observed that 3 h brachial FMD was not affected following a single dose of 8 mmol of nitrate salt in healthy participants, even though circulating plasma nitrate and nitrite (NOx) concentrations were still elevated. Similar results have been observed in this study since nitrate plus nitrite concentrations remained elevated 4 h post-drink while no effects on FMD were found. Thus, it is possible that inorganic nitrate significantly improves FMD values up to two hours, but not 3 and 4 h responses irrespective of plasma NOx concentrations that may still be increased. In contrast to the previously mentioned studies, Joris and colleagues [17] provided a high-fat mixed-meal next to the beetroot juice providing 500 mg of dietary nitrate. The authors did not observe an effect on 2 h brachial FMD responses in overweight and obese individuals after the beetroot juice ingestion. However, this study revealed that acute beetroot juice ingestion attenuated the postprandial worsening in FMD caused by high-fat meals. Therefore, it is possible that a high-fat mixed-meal challenge is needed to determine the postprandial effects of nitrate on FMD responses.

Acute effects of inorganic nitrate were assessed on both brachial and femoral artery FMD responses. Thijssen et al. [8] already reported that there is no significant association between FMD measurements of the brachial and femoral arteries, although these are both peripheral muscular arteries and are able to increase the production of NO in response to flow stimuli. Measurement of FMD responses at different sites is important, as endothelial function is not uniform along the arterial tree [26]. In fact, blood pressure levels are higher in the femoral artery than those in the brachial artery, which is due to the hydrostatic pressure gradient [27]. In addition, the femoral artery is more subject to arterial stiffening, which occurs to a lesser extent in the brachial artery [28]. As in our study, previous studies have also found lower femoral artery as compared with brachial artery FMD values [29]. Intervention effects between sites may thus be different. We did not observe an effect on brachial artery FMD, nor on FMD of the femoral artery, and therefore strong evidence was provided for the absence of an effect of inorganic nitrate on 4 h endothelial function of peripheral muscular arteries.

CAR responses to a cold pressure test were assessed as a non-invasive measure of endothelial function of a central elastic artery. These responses are strongly related to CVD risk factors and may also predict future CVD events [11,30]. Contrary to FMD, mechanisms underlying the CAR are less well understood. The CAR response to a cold pressure test is determined by the balance between vasodilation and vasoconstriction that are caused by the release of catecholamines, such as norepinephrine [12]. Norepinephrine is known to induce vasodilation via endothelium-dependent effects, whereas it also causes vasoconstriction of smooth muscle cells via effects on the sympathetic nervous system [12,31]. A broad range of CAR responses can thus be expected, which can either be positive or negative, as compared with FMD values that usually only vary between 2% and 8%. As mentioned before, endothelial function is not uniform across all arteries [26]. In fact, the carotid artery is a major central elastic artery that has different properties compared with peripheral muscular arteries [32]. Although the function of both types of arteries is associated with CVD risk, carotid artery function may be more strongly associated with cerebrovascular disease, while the function of muscular arteries may be more strongly related to coronary heart disease [28]. As a result, the focus should be on both central elastic and peripheral muscular arteries as this will provide a more complete picture of the impact on endothelial function throughout the arterial tree. In line with the FMD results, no effects of inorganic nitrate on CAR responses were observed. However, a significant difference in 4 h baseline carotid artery diameters was found with higher diameters after the potassium nitrate drink as compared with placebo. A greater baseline carotid artery diameter may also predict a higher CVD risk [33], but effects should be interpreted with caution as only 4 h carotid artery measurements were performed, and fasting values were not available to calculate changes.

In the present trial, the pFMDv was calculated to correct FMD responses for changes in velocity stimulus. Moreover, the focus was on the time-integrated course of the vasodilatory response. In fact, previous research has shown that changes in the flow stimulus and time to peak dilation explained differences between individual FMD responses [34,35]. Further, the time to peak dilation of the brachial artery may be associated with CVD risk. People with an increased time to peak dilation had an increased CVD risk as compared with those with similar FMD responses but a shorter time to peak dilation [36]. The pFMDv, velocity flow, and time to peak dilation did, however, not differ between the drinks. In agreement, Walker et al. [9] also found no effect of inorganic nitrate after 2 h on the flow stimulus and the time to peak dilation. Further, no effects on the fasting time to peak dilation were observed after 4-week consumption of beetroot juice in patients with hypertension, although FMD was improved [37]. We did however observe an increase over time in brachial pFMDv, but not in femoral pFMDv. This could be explained by an increased 4 h peak velocity flow, which may be related to increased blood pressure levels observed after the drinks [38].

No effects of potassium nitrate were observed on SBP, DBP and HR. Previous studies have shown that the intake of nitrate salt and beetroot juice decreased in healthy young adults with 2 and 3 h SBP, but not 4 h SBP [4,25,39]. DBP and HR were not affected in previous studies. The absence of an effect on blood pressure in our study may thus be due to the timing of the measurements. However, one trial also observed a significant decrease in 4 h SBP after a high dose (i.e., 24 mmol) of potassium nitrate [19], but this dose was more than double the amount provided by us. In addition, no effects of a single dose of 500 mg beetroot juice were observed on SBP in a study with overweight individuals [40], a comparable population compared with ours. This may indicate that the inclusion of overweight or obese individuals may also be responsible for the absence of blood pressure effects. Finally, we found a significant increase in SBP and DBP over time, while HR decreased, which could be related to the circadian clock-mediated regulation of blood pressure [41,42].

A limitation of the present study is that only men were included. This reduces the external validity of our study results but excludes the possibility of any possible sex effects. In addition, participants received an intranasal insulin spray during the test day as described elsewhere [13]. However, serum insulin concentrations remained stable during the test day and were not affected by the drinks. Finally, total NO metabolite concentrations were measured using a colorimetric nitrite/nitrate Griess assay kit that may suffer from analytical issues, which have been discussed elsewhere in detail [16,43]. These problems should be considered when interpreting serum nitrate plus nitrite results.

In conclusion, this randomized controlled, double-blinded, placebo-controlled cross-over study provided no evidence for the beneficial effects of a single dose of inorganic nitrate on 4 h vascular endothelial function in abdominally obese men. Authors should discuss the results and how they can be interpreted from the perspective of previous studies and the working hypotheses. The findings and their implications should be discussed in the broadest context possible. Future research directions may also be highlighted.

## Figures and Tables

**Table 1:** Baseline characteristics of the abdominally obese men who completed the study.

Characteristics	
Age (years)	50 (45–57) ^1^
Body mass index (kg/m^2^)	33.5 ± 5.0
Waist circumference (cm)	117.3 ± 10.3
Fasting serum total cholesterol (mmol/L)	5.1 ± 0.7
Fasting plasma glucose (mmol/L)	5.5 ± 0.6

Values are means ± SDs unless otherwise stated. ^1^ Median (interquartile range).

**Table 2:** Pre-drink, post-drink, and changes in brachial and femoral flow-mediated vasodilation, carotid artery reactivity, and blood pressure outcomes of the eighteen men who completed the study.

Variables		Placebo	Potassium Nitrate
Baseline brachial artery diameter, mm	Pre-drink	4.7 ± 0.5	4.7 ± 0.6
	Post-drink *	4.6 ± 0.6	4.6 ± 0.6
	Changes	−0.1 ± 0.2	−0.1 ± 0.2
Brachial artery pFMDv, %	Pre-drink	2.0 ± 2.0	2.5 ± 3.1
	Post-drink *	1.1 ± 0.8	1.2 ± 0.9
	Changes	−0.9 ± 1.5	−1.3 ± 3.0
Brachial velocity flow, %	Pre-drink	284 ± 116	276 ± 116
	Post-drink *	404 ± 77	370 ± 99
	Changes	120 ± 117	095 ± 139
Brachial time to peak dilation, s	Pre-drink	67 ± 21	82 ± 50
	Post-drink	70 ± 36	60 ± 14
	Changes	3 ± 42	−22 ± 55
Baseline femoral artery diameter, mm	Pre-drink	7.5 ± 0.8	7.5 ± 0.9
	Post-drink	7.6 ± 0.8	7.5 ± 0.8
	Changes	0.0 ± 0.3	0.0 ± 0.3
Femoral artery pFMDv, %	Pre-drink	1.0 ± 0.9	1.1 ± 0.9
	Post-drink	1.3 ± 1.2	1.1 ± 1.2
	Changes	0.3 ± 1.2	0.1 ± 1.7
Femoral velocity flow, %	Pre-drink	260 ± 62	244 ± 54
	Post-drink	284 ± 63	259 ± 840
	Changes	24 ± 81	15 ± 88
Femoral time to peak dilation, s	Pre-drink	112 ± 57	98 ± 48
	Post-drink	112 ± 53	105 ± 57
	Changes	0 ± 85	7 ± 78
CAR, %	Pre-drink	–	–
	Post-drink	4.0 ± 2.9	3.2 ± 2.5
	Changes	–	–
Baseline carotid artery diameter, mm	Pre-drink	–	–
	Post-drink	7.1 ± 0.5	7.2 ± 0.6 **
	Changes	–	–
SBP, mmHg	Pre-drink	128 ± 15	128 ± 12
	Post-drink *	132 ± 13	132 ± 15
	Changes	4 ± 7	3 ± 7
DBP, mmHg	Pre-drink	81 ± 9	81 ± 8
	Post-drink *	85 ± 8	84 ± 9
	Changes	4 ± 4	3 ± 5
HR, beats/min	Pre-drink	62 ± 9	61 ± 8
	Post-drink *	56 ± 8	57 ± 6
	Changes	−5 ± 4	−5 ± 4

Values are means ± SD. CAR: carotid artery reactivity; DBP: diastolic blood pressure; HR: heart rate; pFMDv: flow-mediated vasodilation response corrected for peak velocity stimulus; SBP: systolic blood pressure. * *p* < 0.05 significantly different from fasting values; ** *p* < 0.05 significantly different from placebo.

## Data Availability

De-identified data may be shared when possible and made available upon reasonable request to the corresponding author and subject to an approved proposal and data access agreement.

## Supplementary Materials

The following supporting information can be downloaded at: https://www.mdpi.com/article/10.3390/nu14173560/s1, Figure S1: Schematic overview of study design [13]; Figure S2: The CONSORT flow diagram of the volunteers screened, included and analyzed in this randomized trial; Figure S3: Mean serum nitrate concentrations (±SD) after the potassium nitrate and placebo drink [13]; Table S1: Fasting total cholesterol, high-density lipoprotein cholesterol, low-density lipoprotein cholesterol, triacylglycerol, glucose and high-sensitivity C-reactive protein concentrations of the eighteen abdominally obese men who completed the study.
