# Supplementary material for: Acute Effects of Inorganic Nitrate Intake on Brachial and Femoral Flow-Mediated Vasodilation, and on Carotid Artery Reactivity Responses: Results of a Randomized, Double-Blinded, Placebo-Controlled Cross-Over Study in Abdominally Obese Men
Source: Nutrients. 2022 Aug 29;14(17):3560. doi: 10.3390/nu14173560 (PMC9460748; doi:10.3390/nu14173560)
Supplement: Supplementary file 1 [file nutrients-14-03560-s001.zip › nutrients-1841284-supplementary.pdf]

Supplementary Materials

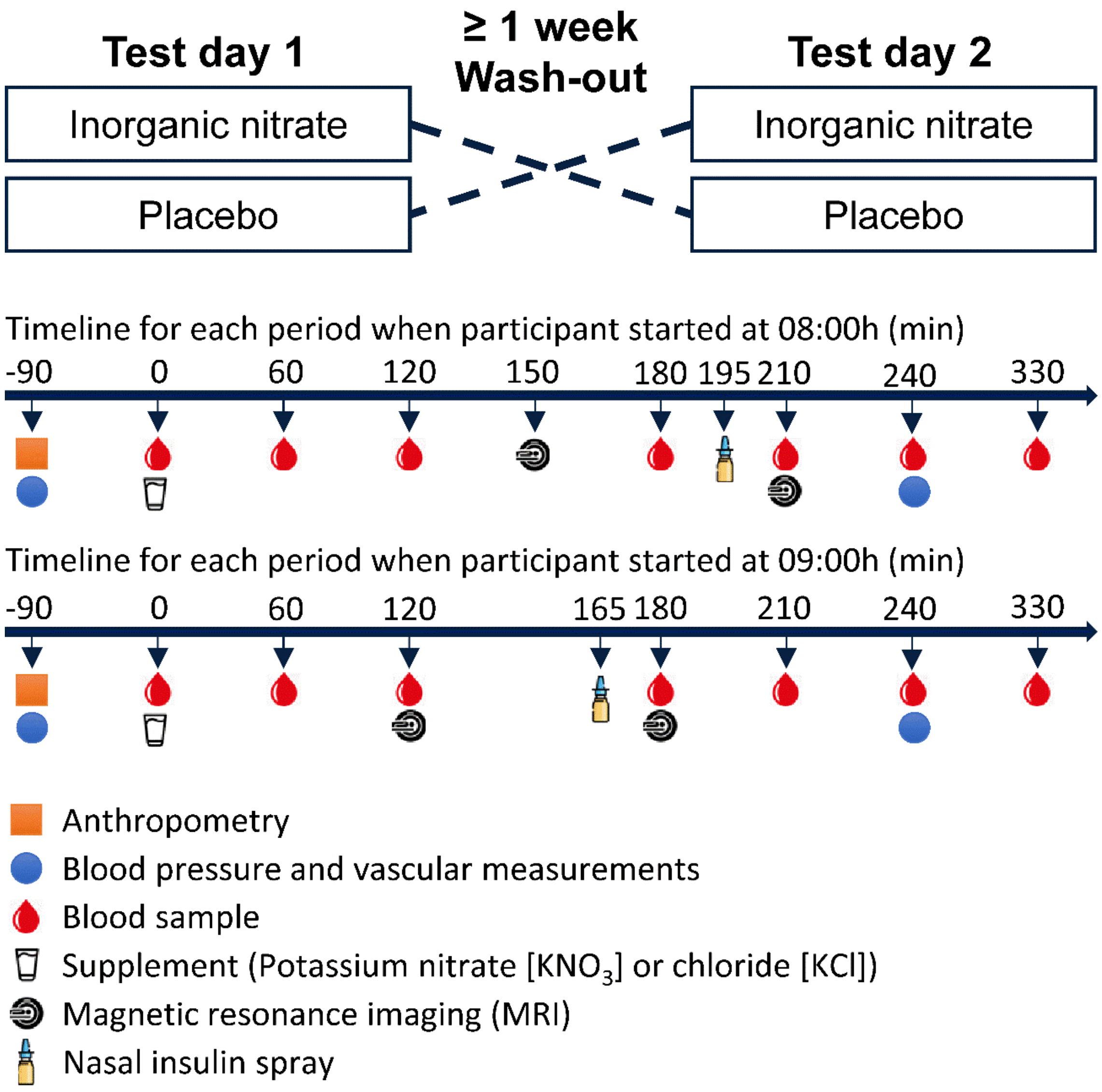

Figure S1. Schematic overview of study design [13].

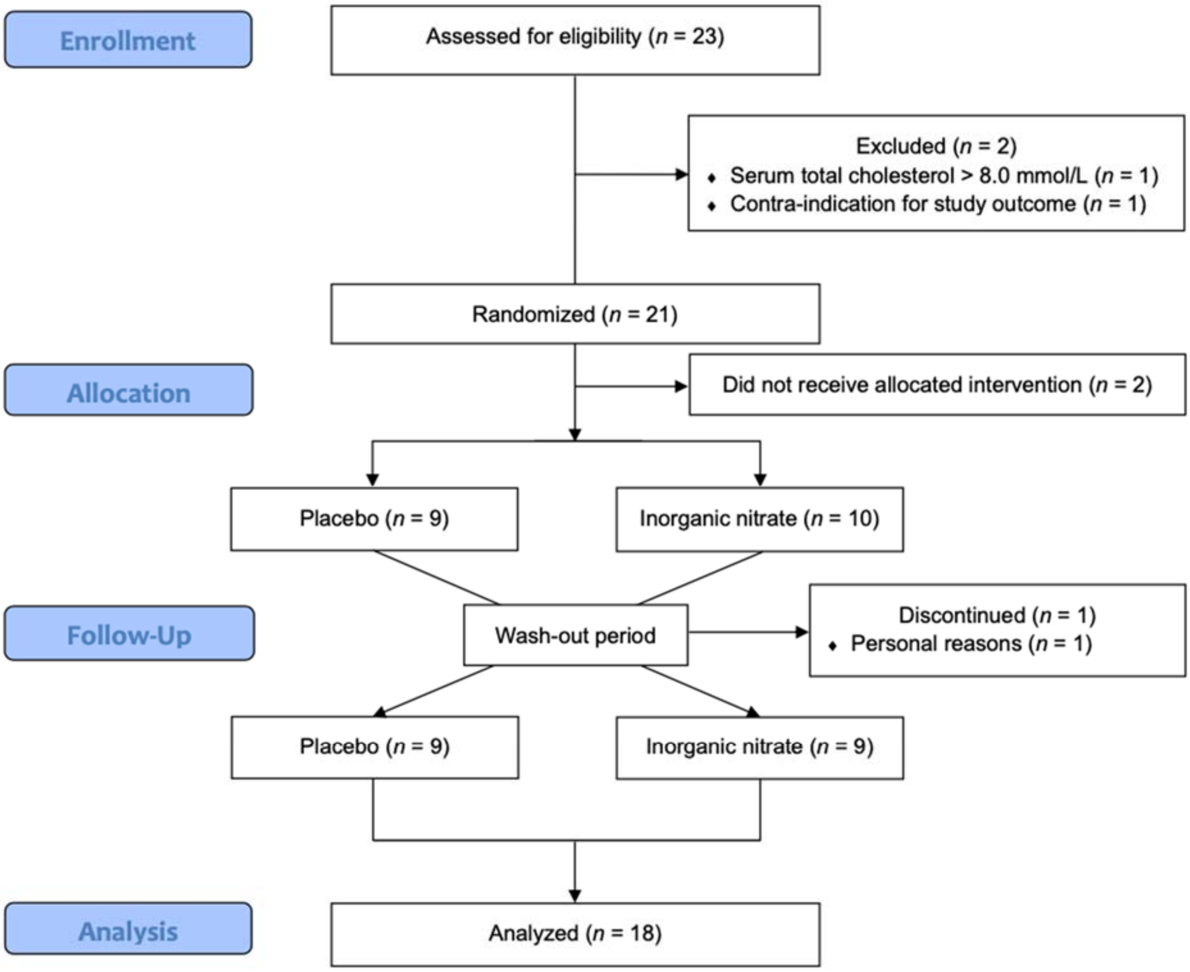

Figure S2. The CONSORT flow diagram of the volunteers screened, included and analyzed in this randomized trial.

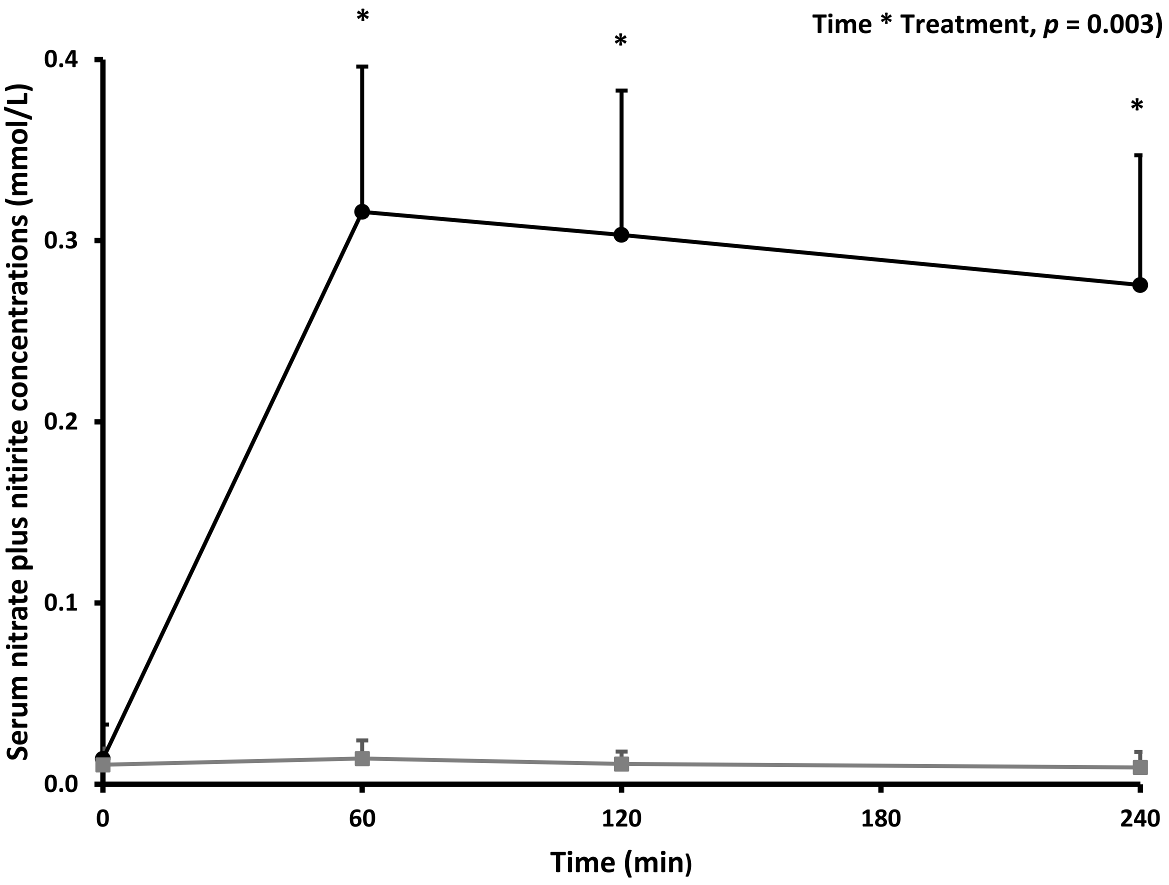

Figure S3. Mean serum nitrate concentrations (± SD) after the potassium nitrate (●) and placebo drink (■; $n = 18$). * Post-drink serum nitrate plus nitrite concentrations were higher at T60, T120 and T240 after consumption of the inorganic potassium nitrate drink ($p < 0.001$, for all time points) [13].

Table S1. Fasting total cholesterol, high-density lipoprotein cholesterol, low-density lipoprotein cholesterol, triacylglycerol, glucose and high-sensitivity C-reactive protein concentrations of the eighteen abdominally obese men who completed the study.

**Variables**	**Placebo**	**Potassium nitrate**
Total cholesterol (mmol/L)	5.0 ± 0.9	4.9 ± 0.7
HDL-cholesterol (mmol/L)	1.0 ± 0.2	1.1 ± 0.2
LDL-cholesterol (mmol/L)	3.7 ± 0.8	3.5 ± 0.6
TAG (mmol/L)	1.3 ± 0.8	1.4 ± 0.6
Insulin (IU)	9.3 ± 6.6	7.3 ± 5.7
Glucose (mmol/L)	5.8 ± 0.5	5.8 ± 0.6
hsCRP (mg/L)[1]	1.3 (0.8 – 4.4)	1.2 (0.8 – 3.6)
Nitrate plus nitrite (mmol/L)	0.01 ± 0.01	0.01 ± 0.02

Values are means ± SDs unless otherwise stated. HDL: high-density lipoprotein; hsCRP: high sensitivity C-reactive protein; LDL: low-density lipoprotein; TAG: triacylglycerol. [1] Values are median (interquartile range).

References

13. Kleinloog, J.P.D.; Mensink, R.P.; Smeets, E.T.H.C.; Ivanov, D.; Joris, P.J. Acute inorganic nitrate intake increases regional insulin action in the brain: Results of a double-blinded, randomized, controlled cross-over trail with abdominally obese men. *Neuroimage. Clin.* **2022**, *35*, 103115.
